# Supplementary material for: Synchronization, Stochasticity, and Phase Waves in Neuronal Networks With Spatially-Structured Connectivity
Source: Front Comput Neurosci. 2020 Oct 19;14:569644. doi: 10.3389/fncom.2020.569644 (PMC7604323; doi:10.3389/fncom.2020.569644)
Supplement: Supplementary file 1 [file Data_Sheet_1.PDF]

Supplementary Material

1 SUPPLEMENTARY DATA

Tabulated values for $\Phi_\sigma(I)$ and $\tau^{(fat)}(I)$ are provided in a separate data file in text format.

2 SUPPLEMENTARY FIGURES

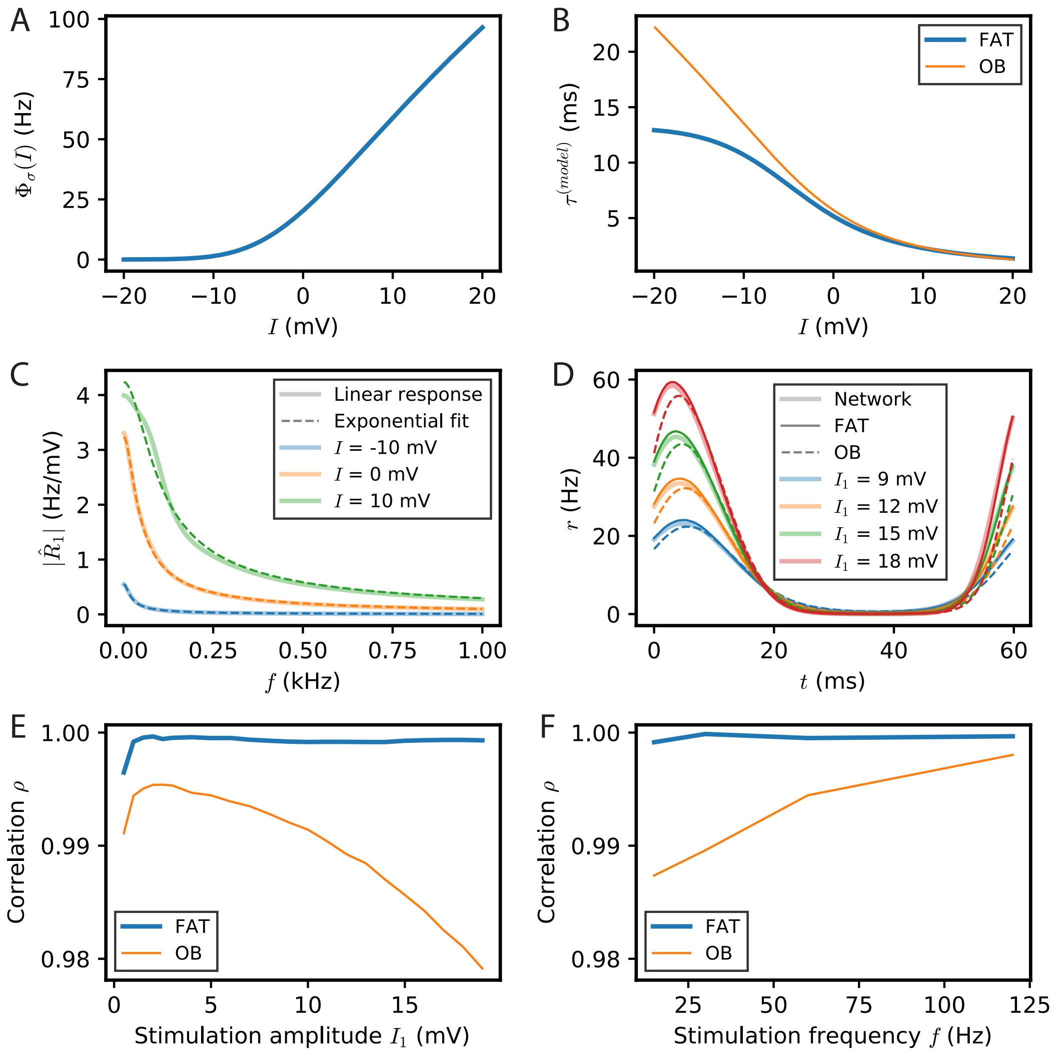

Figure S1. The FAT rate model for EIF neurons. (A) The f-I curve $\Phi_\sigma(I)$ for the EIF neurons used in the rate-model formulation (numerically evaluated following (Richardson, 2007)). (B) The fitted adaptive timescale $\tau^{(FAT)}(I)$ determined by fitting the linear firing-rate response and used throughout the manuscript (thick blue line). For comparison, we also show the adaptive timescale proposed by (Ostojic and Brunel, 2007) (OB, thin orange line). (C) Examples of the fit of the analytically determined firing-rate response with the Fourier transformation of an exponential kernel in the time domain (see *Methods*). (D) Dynamics of a population of (uncoupled) EIF neurons and the two adaptive rate models upon injection of a sinusoidal current $I_1 \sin(2\pi f t)$ for $f = 17\,\mathrm{Hz}$ and different values of I_1. (E,F) Correlation between the network activity and the two adaptive rate models (FAT, thick blue; OB, thin orange) as a function of the amplitude I_1 of the injected current for a fixed frequency $f = 17\,\mathrm{Hz}$ (E), and as a function of frequency f for a fixed amplitude $I_1 = 3\,\mathrm{mV}$ (F).

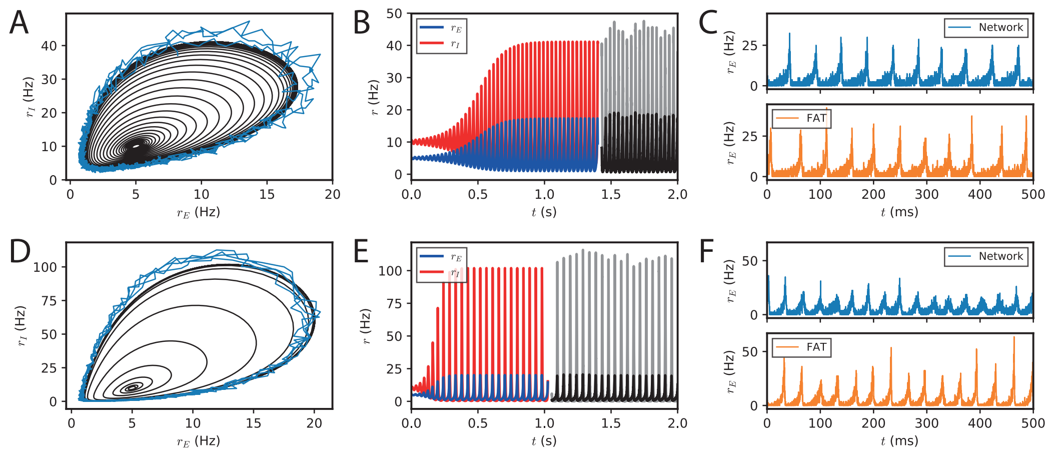

Figure S2. Oscillatory E-I module dynamics for reference parameters B and C. (A,B) The deterministic limit cycle dynamics for a single E-I module described by the FAT rate model with synaptic strengths corresponding to reference case B (see Fig. 1A) is shown together with spiking network simulations with $N = 10^6$. The synaptic strengths are given by$w_{EE} = 1.6\,\mathrm{mVs}$, $w_{IE} = 1.6\,\mathrm{mVs}$, and $w_{EI} = 0.8\,\mathrm{mVs}$. (C) A comparison of the stochastic FAT rate model with spiking network simulations with $N = 10^4$ for the same parameters. (D-E) Analogous plots for the reference case C, with $w_{EE} = 1.76\,\mathrm{mVs}$, $w_{IE} = 3.2\,\mathrm{mVs}$, and $w_{EI} = 0.4\,\mathrm{mVs}$.

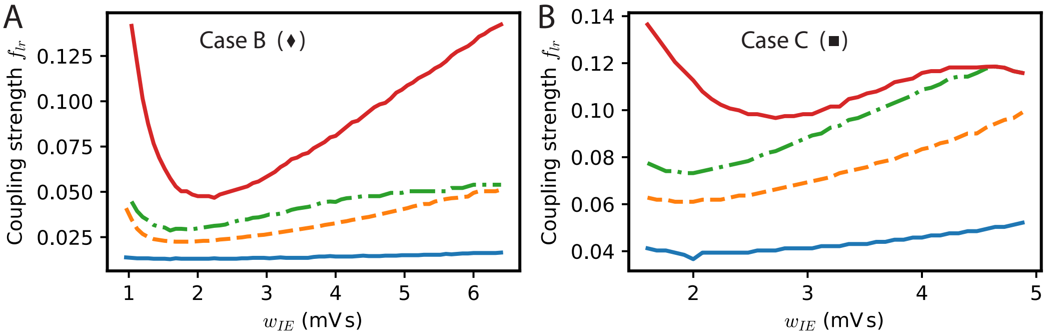

Figure S3. Dynamical regimes of two coupled E-I modules for reference parameters B and C. The analog of Fig. 2B for the two other reference parameters shown on Fig. 1A. The phase diagrams for case B ($w_{EE} = 1.6\,\mathrm{mVs}$, $w_{IE}w_{EI} = 1.28\,\mathrm{mV^2s^2}$) and case C ($w_{EE} = 1.76\,\mathrm{mVs}$, $w_{IE}w_{EI} = 1.28\,\mathrm{mV^2s^2}$) are shown on panels (A) and (B), respectively.

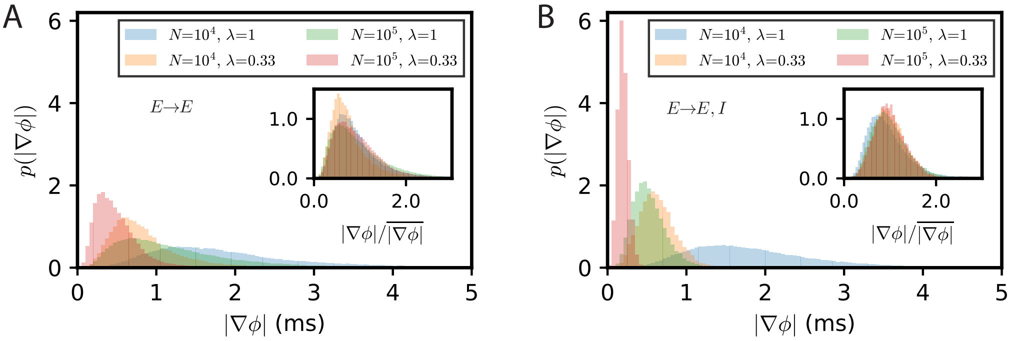

Figure S4. Average phase gradients for a chain of E-I modules. The probability distribution of extended phase gradients $\overline{\nabla\phi_x}$ that exceed the standard deviation of locally contributing phase differences (see *Methods*), for different values of λ and N, for $E \to E$ connectivity (A) and $E \to E, I$ connectivity (B).

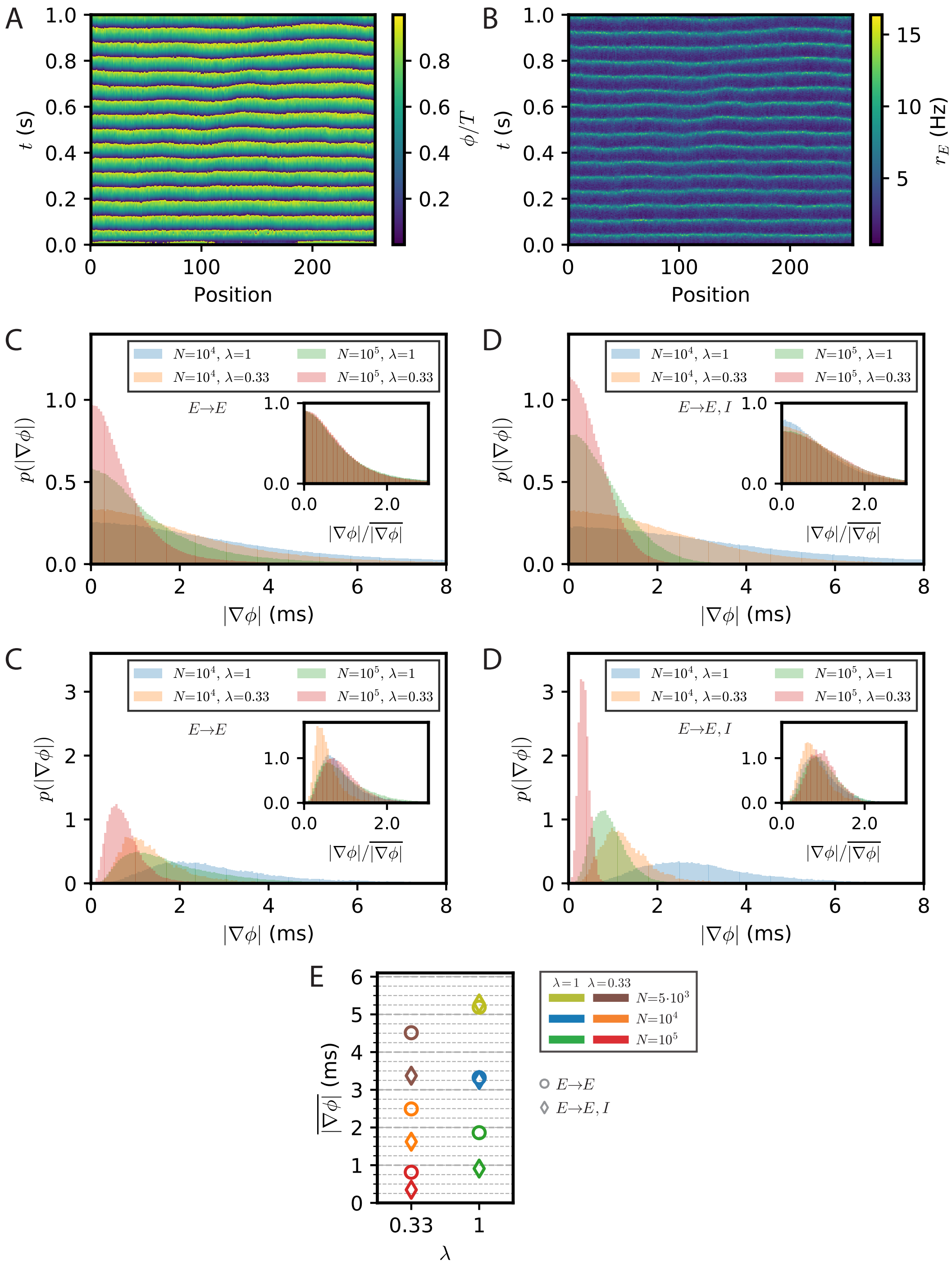

Figure S5. Phase gradients in a chain of E-I modules with decreased long-range excitation. (A,B) Simulation of the stochastic rate model (FAT) for a chain with $E \to E, I$ connectivity, a space constant $\lambda = 0.33$, and network size $N = 10^4$. In contrast to Fig. 7D,E, the contribution of the long-range connectivity to the total recurrent excitatory drive is lowered by a factor 2 at the expense of purely local recurrent excitation. (A) Instantaneous phase as obtained from the Hilbert transform of bandpass-filtered excitatory activity, where the (unfiltered) rates are shown in (B). (C,D) Histograms of the local phase differences for different values of λ and N for $E \to E$ connectivity (C) and $E \to E, I$ connectivity (D). (E,F) Histograms of the extended phase gradients $\overline{\nabla\phi}$. The averages of the distributions shown in (E,F) are plotted in (G), and are the analog of Fig. 7I for a diminished contribution (by a factor of 2) of long-range connectivity to the total recurrent excitatory drive.
